# Gas gangrene following implant removal after the union of a tibial plateau fracture: a case report

**DOI:** 10.1186/s12891-018-2186-4

**Published:** 2018-07-25

**Authors:** Shanxi Wang, Lei Liu

**Affiliations:** Department of Orthopedics, West China Hospital, Sichuan University, 37# Guoxue Alley, Chengdu, 610041 People’s Republic of China

**Keywords:** Gas gangrene, Implant removal, Complication, Amputation

## Abstract

**Background:**

Gas gangrene is an invasive, fatal anaerobic infection that is characterized by acute, rapid evolution and high mortality. Gas gangrene is often secondary to open fractures with deep wounds but is extremely rare in the patients undergoing elective surgery. Implant removal is a common elective operation in orthopedics after the union of fractures, and the complications of this surgery include infection, nerve injury and re-fracture. However, to the knowledge of the authors, there is no report in the literature on gas gangrene following implant removal. Here, we present a case study of gas gangrene following the removal of an internal fixation device after the union of a tibial plateau fracture.

**Case presentation:**

A 59-year-old man with a postoperative union of a left tibial plateau fracture after open reduction and internal fixation complained of severe pain in the wound region on the first morning after implant removal surgery, and the incision was severely swollen and filled with hemorrhagic content. On the second morning, the patient’s symptoms were aggravated progressively. The patient experienced delirium on the third morning after surgery, and a physical examination revealed subcutaneous crepitus extending along the length of the limb, and roentgenograms revealed the accumulation of gas in soft tissue. Gas gangrene was highly suspected, and the left femoral amputation was performed the following night at approximately 11 p.m. General supportive therapy and antibiotic therapy were given subsequently, and the patient was recovered and discharged after his vital signs were stable.

**Conclusions:**

Although gas gangrene is rare, its high mortality and disability indicate that we should pay attention to its prophylaxis, and strict aseptic techniques should be emphasized for even the most minor procedure.

## Background

Gas gangrene is an invasive, fatal anaerobic infection caused by Clostridium, especially *Clostridium perfringens*, that is often secondary to open fractures, deep wounds, and other injuries. Gas gangrene is characterized by acute, rapid evolution and high mortality. The clinical symptoms of gas gangrene include swelling and necrosis of massive muscles, accumulation of gas at the site of infection and other general symptoms, such as fever and sudden onset of prominent pain [1, 2].

Implant removal is a common operation in orthopedics after the union of fractures, especially in weight-bearing bones, and the complications of this surgery include infection, nerve injury and re-fracture [3]. However, to the knowledge of the authors, there is no report in the literature on gas gangrene following implant removal after the union of fractures.

The aim of this paper was to present a patient with gas gangrene following the removal of an internal fixation device at the Department of Orthopedics, West China Hospital, Sichuan University.

## Case presentation

**Fig. 1:**
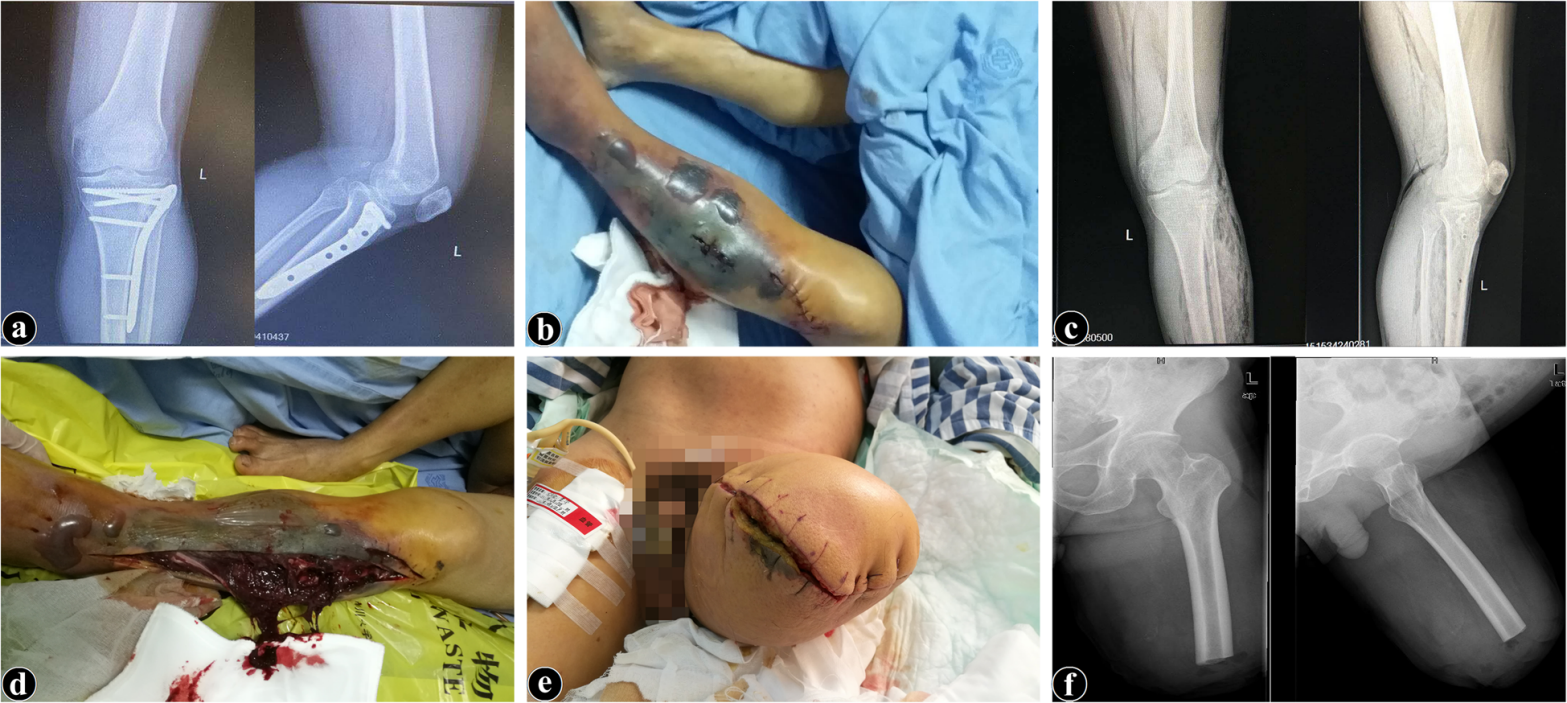
**a** Preoperative X-ray suggesting a bony union of a left tibial plateau fracture. **b** The skin around the wound was darkened, and tension blisters were visible. **c** Postoperative X-ray revealing the accumulation of gas in soft tissue. **d** After incision decompression, the musculature was found to be extensively necrotic and the secretions attached to necrotic muscles were dirty brownish and irritating. **e** Left thigh stump after amputation of the left thigh in the middle third. The wound was left open after meticulous hemostasis. **f** X-ray after the left femoral amputation

The patient is a 59-year-old male with a postoperative union of a left tibial plateau fracture who, after open reduction and internal fixation, underwent surgery for implant removal at a local hospital (Fig. 1a). The first day after surgery, at approximately 9 a.m., he complained of severe pain in the wound region and developed a fever in which the highest temperature reached was 39 °C. Upon examination, the patient was conscious without chills and other discomfort, and the incision was severely swollen and filled with hemorrhagic content. Analgesic therapy was given, but the patient did not feel pain relief. Early on the second morning, the patient started to become irritable and apathetic and the incision was inflamed. The following afternoon, at approximately 3 p.m., the patient felt increasing pain out of proportion to the physical findings accompanied by progressive swelling, numbness, and weakness of the limb. The skin around the wound was darkened, and tension blisters were visible (Fig. 1b). The patient was delirious on the third morning after surgery, the systemic skin turned yellow, and there was a large amount of dark red, bloody discharge drainage from the incision with bubbles of gas, and a foul, sweetish, unpleasant odor was smelled in the incision. A physical examination revealed subcutaneous crepitus extending along the length of the limb, roentgenograms revealed the accumulation of gas in soft tissue (Fig. 1c), the lower limb skin temperature decreased, the dorsal artery of the foot could not be touched, and the patient had no response to pain in the lower extremities. Anaerobic infection gas gangrene was highly suspected, and the patient was transferred to our hospital at approximately 7 p.m.

Left lower extremity incision decompression was carried out immediately. Upon incision, the musculature was found to be extensively necrotic and crepitant, the appearance of the affected muscles was purplish red and brown with a characteristic foul odor, there was an absence of bleeding from the divided surface, and the musculature failed to contract on transverse section. Also, the secretions attached to necrotic muscles were dirty brownish and irritating (Fig. 1d). The smear of secretions revealed more bulky Gram-positive bacilli. Subsequently, a left femoral amputation was performed the following night at approximately 11 p.m., and the stump was left open after meticulous hemostasis (Fig. 1e and f). At the same time, general supportive therapy (fluid resuscitation, blood transfusions, plasmapheresis) and antibiotic therapy (penicillin) were given to stabilize the patient’s vital signs intraoperatively and postoperatively. After surgery, the stump was dressed regularly, and the stump was closed when the condition of the patient improved and the culture of the discharge from the granulating stump showed an absence of gas bacilli. Finally, with the follow-up treatment, the patient recovered and was discharged 2 weeks after left femoral amputation.

## Discussion

Gas gangrene is an invasive, fatal anaerobic infection that is frequent in war. The occurrence of gas gangrene is to be feared in deep wounds complicated by impairment of the main or collateral circulation and especially by extensive crushing of muscle tissue associated with compound fractures. Gas gangrene is most frequently found in wounds of the lower extremity. The period of invasion after trauma is one to 4 days, usually 24 to 48 hours [4].

The common pathogens of gas gangrene are Clostridium, are widely distributed in anaerobic nature and are often found in soil putrilage as well as the digestive tract of animals and humans [5, 6]. Clinically, gas gangrene is usually secondary to deep wounds contaminated with anaerobic Clostridiums, such as open fractures, lacerations, abrasions and burns and may even appear in ulcers. Gas gangrene rarely occurs in patients undergoing elective surgery; only a few cases have been reported in the literature, and this type of gas gangrene is often associated with impaired immune function and the patient usually has a combination of other diseases, such as uncontrolled diabetes mellitus and various forms of malignancies [7–9]. To the knowledge of the authors, there is no report the literature on the gas gangrene following implant removal after the union of fractures. In our case, the patient had no other underlying disease and it was difficult to identify the cause of gas gangrene. After analysis, we believe that gas gangrene may be related to the following aspects. First, the digestive tract of animals and humans is the reservoir of Clostridium. During the perioperative period, the immune function of the patient is low, it is easily to cause an intestinal flora disorder, and the incision can be infected by Clostridium through hematogenous spread. Second, during surgery, application of a tourniquet and injuries to blood vessels and soft tissue can make the muscle inactive, thus creating a hypoxic environment within the incision that is beneficial to the reproduction of Clostridium. Third, too-tight compression of dressing postoperatively may also be a factor that contributes to the occurrence of gas gangrene.

The diagnosis of gas gangrene is mainly based on clinical symptoms and indications. The special sign of gas gangrene is pain in the region of a wound within a period of one to 4 days after injury. This pain is sudden in onset and frequently much more intense than the condition of the wound would indicate. The skin is edematous and darkened, and sometimes, tension blisters are visible around the wounds with a foul odor. There is a characteristic discharge with bubbles of gas, liquefied fat, and spreading crepitation that can be detected within a few hours. Meanwhile, the general condition of patients will deteriorate sharply and result in systemic toxemia, hypotension, shock, multi-organ failure, and death if not controlled [1, 10].

For the treatment of gas gangrene, early and prompt diagnosis cannot be overestimated so that the appropriate treatment can be carried out in a timely manner, including the principles of supportive measures, antimicrobial therapy, and surgical intervention [1, 9–11]. The purpose of systemic supportive therapy (infusion solutions, blood transfusions) is to maintain the stability of the circulatory volume and to improve the general condition of the patient. In the meantime, the use of antibiotics (penicillin, clindamycin, metronidazole) is an important method to combat the effects of the infection [1, 10]. Hyperbaric oxygen therapy can also be considered to be an adjunctive measure, although its effectiveness has not yet been established. Although many methods have been used to treat gas gangrene, surgery is still the most effective method. Surgical intervention includes extensive debridement and amputation, and the type of surgery depends on the affected region, extent of the infection and experience of the surgeon. In general, if only one muscle or muscle group is affected or the infection is on the trunk, these regions should be opened by generous incisions and the subcutaneous tissue and fascia should be removed to reduce tension and establish a broad drainage. On the other hand, if all of the muscles are affected on one extremity, amputation is imperative [1, 2, 12]. The indication for amputation includes extreme lacerations of soft parts, the involvement of the infection on several groups of muscles, the presence of extensive comminution of bone with or without opening into a large joint where gangrene is self-evident, and early development of symptoms of toxemia. Amputation is also indicated when the infection shows a tendency to extend rapidly toward the trunk [1]. When it to sacrifice of the extremity is inevitable, it is important to assess the plane of amputation, which should be well above the infected area. All of the involved muscle tissue and suspected infected soft tissue should be excised, and the stump should be left open to establish a broad drainage until the culture of the discharge from the granulating stump shows an absence of gas bacilli and the infection is under control.

## Conclusion

Although gas gangrene is rare, the high mortality and disability due to gas gangrene force us to pay attention to its prophylaxis, and strict aseptic techniques should be emphasized for even the most minor procedure. For the treatment of gas gangrene, early and prompt diagnosis is critical, which can sometimes determine whether the patient dies or not. Once the diagnosis is made, the appropriate treatment should be carried out, including the principles of supportive measures, antimicrobial therapy, and surgical intervention.
